# Reconciling Magnetically Induced Vertigo and Nystagmus

**DOI:** 10.3389/fneur.2015.00201

**Published:** 2015-09-15

**Authors:** Omar S. Mian, Paul M. Glover, Brian L. Day

**Affiliations:** ^1^School of Applied Sciences, London South Bank University, London, UK; ^2^Sir Peter Mansfield Imaging Centre, School of Physics and Astronomy, University of Nottingham, Nottingham, UK; ^3^Sobell Department of Motor Neuroscience and Movement Disorders, Institute of Neurology, University College London, London, UK

**Keywords:** vestibular, vertigo, nystagmus, magnetic fields, Lorentz force

It has long been known that dizziness and vertigo can sometimes be experienced in and around the high-strength magnetic fields of magnetic resonance imaging (MRI) scanners. Three early mechanistic proposals by which magnetic fields may induce vertigo via stimulation of the vestibular system were dependent on head movement, time-varying magnetic fields, and magnetic field spatial gradients, respectively (1, 2). Although these factors might have a role to play, it has recently become clear from both human and animal studies that they are not necessary to achieve strong magnetic vestibular stimulation: see review by Ward et al. (3). For example, a person lying still in a strong homogenous magnetic field in darkness will experience robust, persistent nystagmus that is dependent on an intact vestibular system (4). The mechanism that was proposed (4) to account for this submits that magnetic fields interact with spontaneous ionic current flowing in labyrinthine endolymph to induce Lorentz forces strong enough to deflect semicircular canal cupulae (4, 5). With this mechanism, a stationary head in a magnetic field will receive vestibular input analogous to a constant angular acceleration, and a person being moved into a magnetic field (such as during patient entry into an MRI scanner) will receive an input akin to a ramp angular acceleration (6, 7). In addition to nystagmus, most individuals will also perceive apparent body rotation when exposed to a 7 T static magnetic field in darkness (4, 8). However, there are inconsistencies between this perception and the induced nystagmus that question whether the two are caused by the same mechanism. The purpose of this article is to discuss these apparent discordances and to put forward arguments that allow for a common mechanism. The article condenses arguments made previously (8) and extends them in light of more recent observations (9).

We refer to observations made in a series of recent studies involving exposure of human participants to the magnetic field inside the bore of 7 T MRI scanners with vision occluded (4, 7–9). Only the static magnetic field was present (no imaging sequences were run). Unless otherwise stated, the participants were lying supine and stationary on a scanner bed, with Reid’s plane (the plane formed by the external auditory meatus and the lower orbital margins) approximately vertical, and orthogonal to the long axis of the body. We refer to this as the neutral head position. Participants were entered into the bore head first and the magnetic field was in a head-to-toe direction.

The first two differences pertain to the commencement and duration of responses. But as will be noted, the differences are not unusual and similar observations are made during real head rotations. First, when participants were slowly and continuously pushed into a 7 T magnetic field, nystagmus became apparent at 1.7 T on average while perception was not reported until 5.1 T (8). It should be pointed out that this apparent difference may be an overestimate of any true threshold differences because of the method used to introduce the participant to the magnetic field. Self-motion perception, in contrast to nystagmus, lags vestibular stimulation by some seconds (10), thus contributing to an apparent higher threshold when continuously moving through the magnetic field gradient. Nevertheless, in rotating-chair experiments, the angular acceleration threshold for perception has been reported to be more than twice that of nystagmus (11). Second, nystagmus persisted for the duration of exposure with only partial decline (4, 7–9), whereas perception typically disappeared after about a minute (4, 8). Similarly, when rotated at constant angular acceleration, perception of rotation lasts for around a minute, while nystagmus persists throughout stimulation (12, 13). The likely explanation for the relatively transient nature of perception and the partial decline of nystagmus is that they are due to central adaptation to continuous vestibular input. Strong support for this comes from the aftereffects induced by removal of the stimulus. In both cases (magnetic and rotation), nystagmus reverses direction and perception of rotation reemerges in the opposite direction to that experienced at the start (4, 8, 12, 13). Reversal of perception and nystagmus upon withdrawal from the magnetic field do not occur when exposure is of very short duration (4, 8), i.e., when adaptation is not given opportunity to occur. A difference in threshold for emergence of nystagmus compared with vertigo may explain the difference in persistence of the two phenomena. That is, a declining (adapting) signal will pass through a higher threshold of vertigo before it reaches a lower threshold of nystagmus resulting in a shorter duration of effect. An alternative and not mutually exclusive possibility is that nystagmus and perception of body rotation are subject to different central adaptation processes. Other temporal aspects (i.e., velocity storage) of vestibularly induced behavior have been suggested to be under the control of only partially overlapping networks for nystagmus and self-motion perception (10).

The third (and more challenging) discordance pertains to the spatial properties of nystagmus and vertigo. In discussing these properties, we refer to the coordinate system of Figure 1A^1^. At an approximately neutral head position, healthy individuals tend to exhibit robust horizontal (rotation about *Z*) nystagmus with little to no vertical (rotation about *Y*) or obvious torsional (rotation about *X*) nystagmus (4, 7–9). In contrast, at this same head orientation, vertigo is typically dominated by perception of rotation about the earth-vertical axis (*X*; i.e., as if the bed were spinning in the earth-horizontal plane) (8). Thus, the ocular and perceptual responses suggest orthogonal vestibular signals (Figure 1B). We suspect this discrepancy is because neither vertigo nor nystagmus is providing a true reflection of net vestibular input.

**Figure 1:**
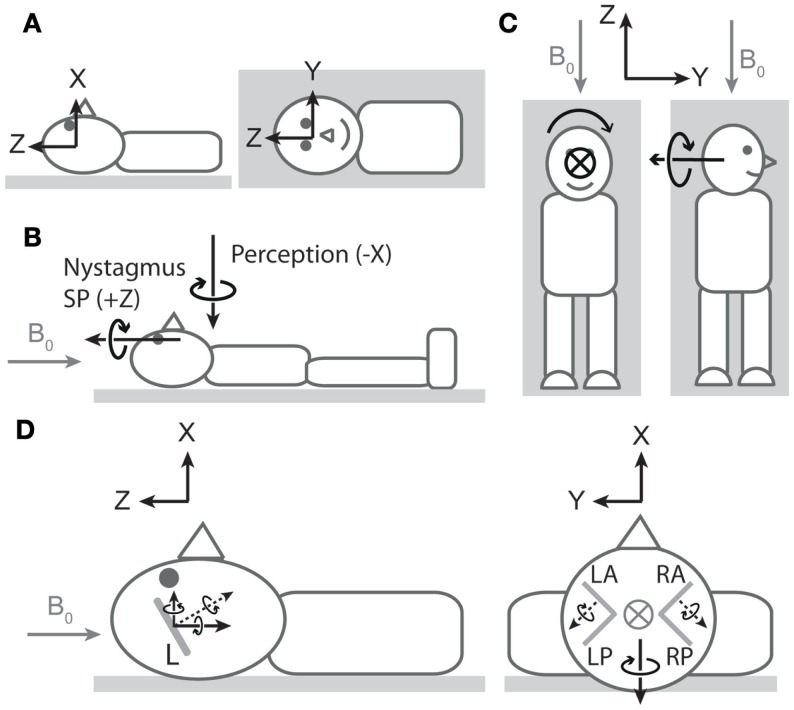
**Nystagmus, vertigo, and hypothetical canal signals during magnetic field exposure**. In all cartoons, except the right side of **(C)**, the head is in the neutral position described in the main text. All vectors are of arbitrary lengths and arbitrary relative lengths. Circle with cross denotes arrow entering page. *Gray arrows* denote magnetic field direction. **(A)** Coordinate system viewed from −*Y* (left cartoon) and +*X* (right cartoon) axes^1^. To describe direction of rotations, we use the right-hand grip rule (point thumb of right hand in direction of axis, and your fingers curl in the direction of positive rotation). Thus, a leftward eye rotation is +*Z*, and a perception of rotation in which the legs rotate to the right in the earth-horizontal plane is −*X*. **(B)** Vectors representing dominant components of nystagmus slow phase (SP) and rotation perception upon exposure to head-to-toe directed magnetic field are orthogonal. The nystagmus SP is +*Z* in most participants, and the perception of rotation is −*X*. The earth-horizontal location of the perception vector is arbitrary^2^. **(C)** Hypothetical rotation signal responsible for perception of rotation about *X* (left) should signal perception of rotation about *Y* when head orientation is altered by 90° about *Z* (right). **(D)** Hypothetical canal signals viewed from −*Y* (left) and +*Z* (right). *Gray lines* within the head are oversize representations of the approximate orientations of lateral (left cartoon) and vertical (right cartoon) semicircular canals (L = lateral, LA = left anterior, LP = left posterior, RA = right anterior, RP = right posterior). *Dotted black arrows* are rotation vectors from the canals inferred from nystagmus SP responses. *Solid black arrows* within the head are components of the vectors in the coordinate system. Left: horizontal eye movements with a leftward SP (+*Z*) imply a rotation vector from lateral canals with −*Z* component^3^. Since the lateral canals are inclined by approximately 20°–25° (14, 15), it also has a +*X* component. This is incongruent with the −*X* perception of rotation at this head position. Right: excitatory rotation signal from the left anterior canal and inhibitory signal from the right anterior canal [hypothesized in Ref. (9)]. The *Y* components of these signals would cancel, but the *X* components would sum leading to a −*X* signal that is directionally congruent with perception of rotation.

With regard to vertigo, the relative lack of perception of rotation about *Z* could be due to conflicting veridical sensory signals (e.g., from otoliths and cutaneous receptors) indicating the body is not rotating in the vertical plane, thus blocking the formation of perception of rotation about an earth-horizontal axis and leading to spatial bias in the perception. To test this idea, we performed an experiment (8) where we reoriented head position by 90° about *Z* (Figure 1C). This does not alter the orientation of the magnetic field with respect to the head and thus should not alter the vestibular signal induced by the magnetic field. The signal responsible for clear perception of rotation about the earth-vertical axis (*X*) in the neutral head position should now signal rotation about *Y* (earth-horizontal) in the new head position. However, this failed to produce perception of rotation about *Y* (8), supporting the notion of spatial bias in the perceptual response. With regard to nystagmus, it is well established that the gain of the torsional vestibulo-ocular reflex (VOR) is considerably lower than that of both horizontal and vertical VOR. The often stated figure is that torsional VOR gain is about 50–60% of horizontal and vertical VOR gains, which has been established during relatively high frequency (0.3–1 Hz) sinusoidal rotations and short transient rotations (16–18). However, during very low-frequency (0.05 Hz) rotations (probably of more relevance for the Lorentz force mode of action) about earth-vertical axes, the gain for torsional VOR was only 20% of horizontal and vertical VOR (17). As a consequence of these gain differences, when the head rotation vector has a head-fixed *X* component combined with a head-fixed *Y* or *Z* component, the reflexive 3D eye movement vector does not align with the head rotation vector (16, 18).

Regardless of sensory-perceptual and sensory-motor reasoning for spatial discordance between vertigo and nystagmus, evidence of a viable canal stimulation pattern is still required if they are to be explained by a common mechanism. The presence of robust horizontal nystagmus (ocular rotation about *Z*) implicates the lateral semicircular canals (4). Owing to their tilt with respect to Reid’s plane, output from the lateral canals would also contain a component of rotation about *X* and so contribute to perception of rotation about that axis. However, the polarity of the resultant component would be incompatible with the observed polarity of perception (Figure 1D, left). This suggests that a unifying explanation must involve suitable simultaneous vertical canal involvement. Initial ocular evidence that vertical canals are amenable to magnetic field stimulation was provided by the observation that altering static head roll orientation (i.e., about *X*-axis) induced vertical nystagmus (i.e., about *head-fixed Y* -axis) (4). A more recent study has provided further indication of vertical canal stimulation, this time at the neutral position (9). Unlike in healthy participants at this head position, patients with unilateral vestibular hypofunction exhibited vertical nystagmus (ocular rotation about *Y*). Based on the direction of nystagmus (which differed depending on side of hypofunction), the authors proposed that a head-to-toe-directed magnetic field leads to excitation of the left anterior canal and inhibition of the right anterior canal (Figure 1D, right). When both sides are functioning normally, the pitch component (*Y*) of the combined anterior canal signals would cancel, explaining the absence of vertical nystagmus (ocular rotation about *Y*) in healthy participants. The authors also noted that the roll components (*X*) would sum. However, not highlighted was that the direction of this summed component (−*X*) is compatible with the direction of the perception of rotation. Thus, we propose that a −*X* component arising from anterior canal stimulation contributes to perception and makes a larger contribution than any +*X* component arising from concurrent activation of the lateral canals.

In conclusion, apparent temporal and spatial discordance between magnetically induced vertigo and nystagmus in the cited experiments is not incompatible with a common mode of stimulation. Temporal discordance is in line with known responses to continuous semicircular canal stimulation, and spatial discordance can be accounted for by spatial bias in both perception and eye movements coupled with simultaneous activation of lateral and anterior semicircular canals.

## Conflict of Interest Statement

The authors declare that the research was conducted in the absence of any commercial or financial relationships that could be construed as a potential conflict of interest.

## Footnotes

^1^For eye movements, the coordinate system is head-fixed. We refer to ocular rotations about the *x*-axis (naso-occipital) as torsion, ocular rotations about the *y*-axis (inter-aural) as vertical, and ocular rotations about the *z*-axis (pointing out top of head) as horizontal. Here, we are adhering to conventional nomenclature for horizontal and vertical eye movements, even though in the neutral head position of the experiments under discussion, these eye movements are orthogonal to earth-horizontal and earth-vertical planes. For perception, the *orientation* of the coordinate system is earth-fixed (*X* = earth-vertical; *Y* and *Z* = earth-horizontal) with origin (i.e., center of perceived rotation) within the confines of the body^2^. For the neutral head position (of primary relevance here), the orientations of the perception and head coordinate systems are aligned.

^2^The location of the perceived center of rotation has not been properly established during exposure to the magnetic field. Our preliminary estimation is somewhere between the center of the head and the naval (8).

^3^Because the slow phase of nystagmus is compensatory, we infer a vestibular signal that has opposite polarity to direction of nystagmus slow phase. We assume polarity of perception reflects polarity of the vestibular signal.
